# Transformation of T-lymphoblastic lymphoma into acute myeloid leukemia after recurrence: a case report and literature review

**DOI:** 10.3389/fonc.2025.1673318

**Published:** 2025-12-12

**Authors:** Mengzhuo Yang, Wenmiao Mei, Xiaoqiang Zhao

**Affiliations:** 1 College of Clinical Medicine, The First Affiliated Hospital of Henan University of Science and Technology, Luoyang, China; 2 The Clinical Medical College of the First Affiliated Hospital of Xinxiang Medical University, Xinxiang, China

**Keywords:** T-lymphoblastic lymphoma, acute myeloid leukemia, lineage transformation, therapy-related leukemia, venetoclax

## Abstract

**Objective:**

To investigate the diagnosis and treatment of the condition of T-lymphoblastic lymphoma (T-LBL) transforming into acute myeloid leukemia (AML) following recurrence.

**Methods:**

We conducted a retrospective analysis of a patient admitted to the First Affiliated Hospital of Henan University of Science and Technology in July 2022 with recurrent T-LBL that progressed to AML. Clinical manifestations, genetic and histopathological findings, and treatment outcomes were evaluated, complemented by a literature review.

**Results:**

The patient, initially diagnosed with T-LBL, achieved complete remission (CR) after combination chemotherapy. Following treatment cessation, the disease recurred and transformed into AML.

**Conclusion:**

The transformation of T-LBL into AML is exceedingly rare, and the underlying mechanisms of lineage switch remain unclear. Venetoclax combined with chemotherapy and allogeneic hematopoietic stem cell transplantation (allo-HSCT) may improve prognosis in such cases.

## Introduction

T-lymphoblastic lymphoma (T-LBL) and acute myeloid leukemia (AML) are aggressive hematological malignancies with poor prognosis if untreated. T-LBL arises from immature T-cell precursors and is characterized by high aggressiveness, rapid proliferation, and heterogeneity, leading to poor prognosis if untreated (1). The co-occurrence of T-LBL and AML in the same patient is rare, and transformation from T-LBL to AML is exceptional. We present a case of an adult patient with recurrent T-LBL that transformed into AML, detailing the clinical course, diagnostic findings, and treatment, alongside a literature review.

## Case report

A 60-year-old male presented in July 2022 with a one-year history of T-LBL and recent recurrence over the prior three months. In June 2022, he noted painless enlargement of the left cervical lymph nodes. Neck Doppler ultrasonography revealed multiple solid hypoechoic nodules bilaterally, with no thyroid abnormalities. One month later, nausea and vomiting prompted hospital admission. A left cervical lymph node biopsy confirmed T-LBL, with immunohistochemistry showing positivity for TDT, CD99, CD3 (more +), CD4 (more +), CD34, MPO, and CD8 (less +), and negativity for CD20, PAX5, CD117, CD10, and CD30. The Ki-67 proliferation index was approximately 60%, and CD21 indicated disruption of the follicular dendritic cell network. *In situ* hybridization for Epstein-Barr encoding region (EBER) was negative. Positron emission tomography-computed tomography (PET-CT) revealed lymphoma involvement in lymph nodes of the bilateral neck, clavicular fossa, axilla, chest wall, right inner mammary gland, abdominal cavity, retroperitoneum, pelvic cavity, bilateral inguinal regions, and subcutaneous nodules of both upper arms. Additional findings included pulmonary infections and prior cholecystectomy. The patient was diagnosed with stage IV non-Hodgkin T-LBL with a low-risk International Prognostic Index (IPI) score of 2.

Following resolution of the pulmonary infection, the patient received 3 complete cycles of alternating Hyper-CVAD (Cyclophosphamide, Vincristine, Doxorubicin and Dexamethasone) and MA (Methotrexate and Cytarabine) chemotherapy regimens between August 7, 2022 and January 3, 2023.Subsequent treatments included CE(Cyclophosphamide,Etoposide) regimen with granulocyte colony-stimulating factor and plerixafor for mobilization (February 6, 2023), CHOP(Cyclophosphamide,Doxorubicin,Vincristine and Prednisone) regimen (March 14, 2023), and COPE(Cyclophosphamide, Doxorubicin, Prednisone and Etoposide) regimen (April 21, 2023). After achieving complete remission (CR) following multiple cycles of chemotherapy, the patient discontinued treatment for more than 2 months due to age-related factors and poor treatment adherence, without regular follow-up visits.

In June 2023, the patient experienced recurrent neck lymph node enlargement. A PET-CT scan on June 29, 2023, at the First Affiliated Hospital of Zhengzhou University revealed metabolically active lymph nodes in the left neck (area V), left anterior chest wall, and left axilla, suggestive of lymphoma infiltration. Bone marrow and blood flow cytometry detected a small CD8dim population (1.6%). Lymph node puncture confirmed T-LBL with immunohistochemistry negative for CD20, CD10, PAX-5, and AE1/AE3, and positive for CD3 (partial), CD7, CD33, CD99, CD34 (partial), TDT, MPO (partial), CD43, CD5 (partial), CD8 (minority), CD4 (partial), and CD30 (scattered). The Ki-67 index was approximately 60%, with CD21 indicating follicular dendritic cell disruption and negative EBER. Flow cytometry revealed 0.17% abnormal precursor T lymphocytes and 65.19% mature T lymphocytes, with a CD4/CD8 ratio of 0.79 and no significant abnormalities in CD2, CD3, CD5, or CD7 expression. The diagnosis was T-cell lymphoblastic leukemia/lymphoma with minimal myeloid marker expression (stage IV). High-intensity chemotherapy followed by allo-HSCT or maintenance therapy was recommended, but the patient declined and was discharged.

**2.**On July 16, 2023, the patient was readmitted with progressive bilateral neck lymph node enlargement. On July 20, 2023, an anti-CD30 monoclonal antibody combined with the ICE (Ifosfamide,Carboplatin and Etoposide)regimen was administered, but the response was poor. The patient underwent a bone marrow puncture biopsy on September 20th. On September 25th, the results of the bone marrow puncture showed: significant active bone marrow proliferation, with 60% of primitive cells, and POX was positive. The bone marrow biopsy indicated an increase in blasts and promyelocytes, consistent with acute leukemia.The bone marrow cell morphology showed: The bone marrow was significantly active in proliferation, with the granulocyte component accounting for 8.00%, the erythrocyte component accounting for 9.50% The proportion of granulocytes was reduced, while the proportion of lymphocytes was normal; and the lymphocytes were mature lymphocytes, with protoblasts accounting for 60%, and POX positive/weakly positive accounting for 65%. Given the advanced lymph node involvement and bone marrow findings, the diagnosis was revised to AML, indicating transformation from T-LBL. The patient’s comorbidities (hypertension, diabetes, prior cholecystectomy, and appendectomy), frailty, and poor chemotherapy tolerance complicated treatment. Following the ineffective anti-CD30 and ICE regimen, a regimen of docetaxel and venetoclax was initiated, and the patient continues regular chemotherapy.

## Discussion

Hematopoiesis classically involves differentiation of hematopoietic stem cells into myeloid and lymphoid progenitors. Multilineage expression in hematological malignancies may manifest as: (1) biphenotypic acute leukemia with simultaneous lymphoid and myeloid antigen expression; (2) lineage conversion, where distinct malignancies arise from different lineages, as reported in a T-ALL case relapsing as AML after CD7-CAR T-cell therapy (2); or (3) concurrent independent hematological malignancies.We use the term “transformation” to describe the disease phenotypic shift from T-LBL to AML, primarily based on the following evidence: the partial positivity of myeloid marker MPO already detected at the initial diagnosis of T-LBL, indicating inherent multilineage differentiation potential; the “biphenotypic transition” with concurrent expression of T-lineage and myeloid markers at the relapse stage; and the phenotypic continuity in the final AML stage—AML cells retained T-lineage-related markers (cCD3-, TdT+, CD7++) while myeloid markers (CD33++, MPO+, POX positivity rate of 65%) were completely consistent with those at the relapse stage. Combined with the compact disease progression timeline, these features suggest that lineage transformation may have occurred in a malignant clone of common origin. However, we must emphasize that prior chemotherapy was undoubtedly a key trigger for this transformation process. Therefore, this case can be etiologically classified as therapy-related AML, but its unique clonal evolution pattern makes it a true lineage transformation. Therapy-related leukemia (t-AML) mainly occurs due to gene mutations caused by damage to normal hematopoietic stem cells and immune cells by radiotherapy and chemotherapy (3),with its core pathogenic feature being the emergence of a *de novo* independent myeloid clone that typically does not carry phenotypic markers of the primary lymphoma. T-AML, often linked to breast cancer or lymphoma, is associated with adverse cytogenetics, increased invasiveness, and poorer prognosis compared to *de novo* AML (4). Alkylating agents and topoisomerase inhibitors are implicated in T-AML development (5). CPX-351, a novel liposomal formulation composed of cytarabine and daunorubicin with a fixed molar ratio of 5:1, is generally preferred for the treatment of T-AML patients who can tolerate intensive induction chemotherapy (6). Depending on the age and health of the patient, especially in those unsuited for intensive chemotherapy, the combination of venetoclax with decitabine or azacitidine is an option (7).

T-LBL is highly aggressive, often presenting with mediastinal masses and pleural or pericardial effusions. Approximately 25-30% of adult LBL patients experience relapse or resistance to first-line therapy (8). Currently, due to limited specific therapeutic targets, multi-drug chemotherapy remains the cornerstone of induction therapy. Various treatment options, including cytotoxic chemotherapy, novel targeted drugs, and cell therapy, have emerged for the treatment of AML, which originates from the malignant clonal proliferation of hematopoietic stem cells (9). Relapsed/refractory (R/R) AML is strongly heterogeneous and is linked with poor prognosis, with a 5-year survival rate of approximately 10% (10).

In this case, the patient achieved CR with T-LBL but relapsed and developed AML after treatment cessation. Treatment options for R/R T-LBL include clinical trials, allo-HSCT after reinductionor auto-HSCT for chemotherapy-sensitive patients. Reinduction regimens include high-dose Ara-C-based combinations, Hyper-CVAD, and new drug combinations (1). Nelarabine yields a 50% overall response rate and 36% CR rate in R/R T-ALL/T-LBL (11). Venetoclax-based regimens have shown promise, with CR rates of 60% in T-ALL/T-LBL, including significant tumor reduction and minimal residual disease (MRD) eradication (12). A venetoclax-azacitidine combination achieved CR in four of five R/R T-ALL patients, with three achieving MRD-negative CR (13). Venetoclax with low-dose navitoclax resulted in a 66.7% CR rate (14). In terms of clinical trials, 60 cases of R/R T-ALL/LBL were treated with anti-CD7 CAR (NS7CAR) T cells, with 94.4% of the patients achieving bone marrow CR after 28 days. Among 32 patients with extramedullary diseases, 78.1% experienced remission, of which 56.3% achieved CR and 21.9% achieved partial remission (15). In a trial involving the treatment of eight patients with R/R T-LBL with autologous CD7-CAR T-cell therapy, a three-month response rate of 87.5% was achieved (16).

CD30 is a transmembrane protein expressed by activated, is a key diagnostic and therapeutic marker in lymphoma (17). A study of 34 T-ALL patients found a CD30 positivity rate of 38%. In this case, the patient’s poor response to anti-CD30 and ICE prompted a switch to venetoclax and decitabine. The combination of AML and LBL in patients often leads to a dismal prognosis. emphasizing the need for treatment strategies addressing both lineages. The use of nilarabine combined with Hyper-CVAD has been found to achieve high CR rates in T-ALL and T-LBL cases. The reason for this may be related to the complexity of the disease, which is often accompanied by high-risk chromosomal karyotypes, and more importantly, the difficulty in balancing the lymphatic and medullary systems with the chemotherapy regimen used. It is thus suggested that the treatment plan for such patients should involve a combination that can balance both the lymphatic and medullary systems or focus specifically on AML, which has a poorer prognosis. Nelarabine combined with Hyper-CVAD has achieved CR rates of 87% for T-ALL and 100% for T-LBL, with a 3-year overall survival of 65% (18). For patients achieving CR, early allo-HSCT is recommended. In patients unsuitable for transplantation, fludarabine and cytarabine may target both myeloid and lymphoid malignancies, though treatment must consider age, prior therapies, comorbidities, and cytogenetic profiles.

This rare case of T-LBL transforming into AML highlights the complexity of lineage transformation and the absence of standardized treatment protocols. Venetoclax-based regimens and allo-HSCT offer potential therapeutic avenues, but further research is needed to optimize outcomes.

## Data Availability

The original contributions presented in the study are included in the article/supplementary material. Further inquiries can be directed to the corresponding author.
